# Dual Functional Antibacterial–Antioxidant Core/Shell Alginate/Poly(ε-caprolactone) Nanofiber Membrane: A Potential Wound Dressing

**DOI:** 10.1021/acsomega.4c02510

**Published:** 2024-05-30

**Authors:** Mohammad-Reza Norouzi, Laleh Ghasemi-Mobarakeh, Fabian Itel, Jean Schoeller, Hossein Fashandi, Giuseppino Fortunato, René M. Rossi

**Affiliations:** † Empa, Swiss Federal Laboratories for Materials Science and Technology, Laboratory for Biomimetic Membranes and Textiles, Lerchenfeldstrasse 5, St. Gallen CH-9014, Switzerland; ‡ Department of Textile Engineering, Isfahan University of Technology, Isfahan 84156-83111, Iran; § Department of Health Science and Technology, ETH Zürich, 8092 Zürich, Switzerland

## Abstract

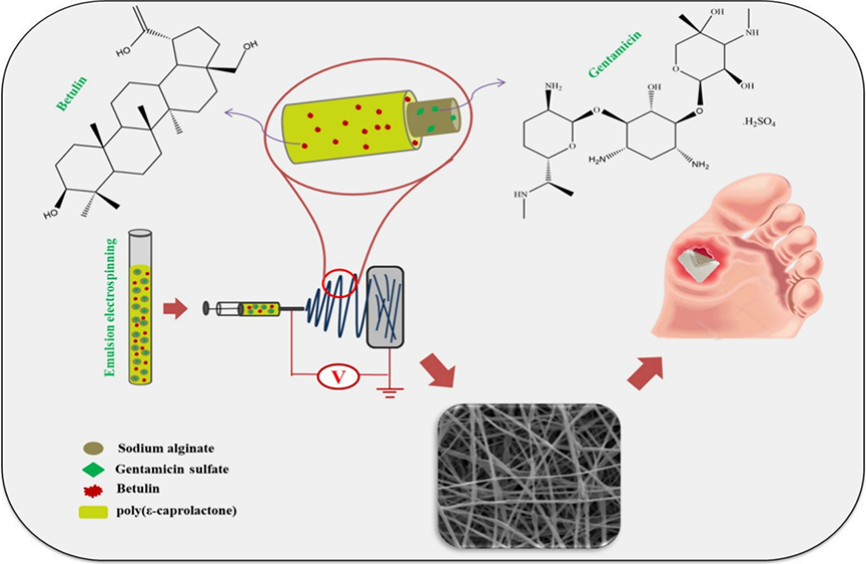

Core/shell nanofibers offer the advantage of encapsulating multiple drugs with different hydrophilicity in the core and shell, thus allowing for the controlled release of pharmaceutic agents. Specifically, the burst release of hydrophilic drugs from such fiber membranes causes an instantaneous high drug concentration, whereas a long and steady release is usually desired. Herein, we tackle the problem of the initial burst release by the generation of core/shell nanofibers with the hydrophilic antibiotic drug gentamycin loaded within a hydrophilic alginate core surrounded by a hydrophobic shell of poly(ε-caprolactone). Emulsion electrospinning was used as the nanofibrous mesh generation procedure. This process also allows for the loading of a hydrophobic compound, where we selected a natural antioxidant molecule, betulin (BTL), to detoxify the radicals. The resulting nanofibers exhibited a cylindrical shape with a core/shell structure. *In vitro* tests showed a controlled release of gentamicin from nanofibers via diffusion. The drug reached 93% release in an alginate hydrogel film but only 50% release in the nanofibers, suggesting its potential to minimize the initial burst release. Antibacterial tests revealed significant activity against both Gram-negative and Gram-positive bacteria. The antioxidant property of betulin was confirmed through the DPPH assay, where the incorporation of 20% BTL revealed 37.3% DPPH scavenging. The nanofibers also exhibited favorable biocompatibility in cell culture studies, and no harmful effects on cell viability were observed. Overall, this research offers a promising approach to producing core/shell nanofibrous mats with antibacterial and antioxidant properties, which could effectively address the requirements of wound dressings, including infection prevention and wound healing acceleration.

## 1 Introduction

The skin is the most frequently injured organ in the human body. Skin wounds usually heal automatically via a complex process. However, prolonged wound healing in chronic wounds has a high risk of severe infections, which can even lead to death-threatening complications if not treated properly.^1^ Gram-positive bacteria such as *Staphylococcus aureus* (*S. aureus*) are the main microorganisms involved during the initial stage of wound infection, while in the later stages of chronic wounds, Gram-negative bacteria such as *Escherichia coli* (*E. coli*) can also be found.^2^ Wound infection leads to the collapse of the granulation tissue and deteriorates the extracellular matrix components and growth factors, which prevent the normal wound healing process.^3^ Moreover, inflammation is a natural response of the body to injury, but if prolonged, it can have negative effects on the wound healing process by delaying the formation of new blood vessels, impairing collagen synthesis, and compromising the immune response, ultimately leading to chronic wounds.^4^ Wide research is being conducted to develop novel materials that can effectively treat wounds in a short period of time. Using a suitable wound dressing incorporated with antibacterial agents for preventing infection and reducing the wound healing time is a common and appropriate treatment approach.^5−7^ However, controlling the antibiotic release profile along with minimum toxicity to local tissue is essential to reach effective infection control.^8^

Pentacyclic triterpenes are natural compounds that are abundant in various parts of plants, such as bark, roots, and leaves.^9^ It has been reported that Lupane-type pentacyclic triterpenes, i.e., lupeol, betulin, and betulinic acid, possess the curative potential through different biological properties, e.g., antioxidant, anti-inflammatory, and antiviral.^10−12^ It has been shown that betulin has a positive influence on cell viability and proliferation and DNA content and promotes the production of sulfated glycosaminoglycans because of its antioxidant activity.^13^ Moreover, researchers have reported that betulin enhances keratinocyte differentiation and accelerates wound healing.^14^

Distinct advantages of nanofibrous wound dressings such as high surface-to-volume ratio render them ideal for drug delivery applications. Moreover, their tunable porosity makes these membranes permeable to gas exchange while, at the same time, inhibiting bacteria penetration.^15−17^ Nanofibrous membranes also mimic the native extracellular matrix, allowing cellular adhesion, proliferation, and differentiation.^18^ Many polymers have been used for the fabrication of nanofibers and the incorporation of different agents such as antibiotics, natural compounds, DNA, and proteins to achieve various controlled release profiles.^19−22^ These drug delivery systems have been used according to two different design concepts, i.e., matrix type and reservoir-type structure. In the former, the active agent is dispersed in the polymeric solution and drug-loaded nanofibers are fabricated through conventional electrospinning, while the latter refers to core/shell electrospun nanofibers where the active agent is trapped in the core of the fibers.^23^ Using two different polymer matrices in the core and shell of the fibers, it is possible to encapsulate multiple therapeutic compounds with different properties within a single carrier. Moreover, the incorporation of a sensitive agent within the core can protect it during the electrospinning process and limit the burst release, which means controlling the release profile of the incorporated agent.^24^ Emulsion electrospinning is a convenient method for the fabrication of core/shell nanofibers using a single needle or even needle-free electrospinning techniques.^25−27^ Several combinations of polymer matrices with core/shell structures have been fabricated using emulsion electrospinning.^28−31^

Poly(ε-caprolactone) (PCL) is an aliphatic polyester extensively used in the biomedical field because of its biocompatibility and appropriate processing.^32^ However, the incorporation of water-soluble compounds in PCL is a challenge due to its hydrophobic nature, which hinders drug–polymer interactions such as hydrogen bonding. Furthermore, the solubility of water-soluble compounds in organic solvents suitable for PCL is another issue.^33^ Sodium alginate (ALG) is a water-soluble biopolymer composed of mannuronic acid and guluronic acid units extracted from algae. ALG has been widely used in biomedical applications due to its nontoxic, biocompatible, biodegradable, and nonimmunogenic properties.^34,35^

The present study aims to prepare antibacterial–antioxidant core/shell ALG/PCL nanofibers for potential wound dressing application. Based on our previous study, where we showed the core/shell fiber formation from the electrospinning of ALG/PCL emulsions, we incorporated gentamicin as a hydrophilic broad-spectrum antibiotic agent and betulin as a hydrophobic natural compound with antioxidant and wound healing properties into ALG and PCL, respectively. The antibacterial and antioxidant properties of the fabricated nanofibrous biomaterial were studied, and the release profile of the antibiotic agent and the radical detoxification of betulin were investigated. Finally, the biocompatibility of this nanofibrous material was assessed.

## 2 Materials and Methods

### 2.1 Materials

Poly(ε-caprolactone) (PCL, *M*_w_ = 80,000), alginic acid sodium salt from brown algae (ALG, low viscosity), chloroform (CHCl_3_), Span 60 (Span), fluorescein sodium salt, betulin (BTL), gentamicin sulfate (GEN), 2,2-diphenyl-1-picrylhydrazyl (DPPH), PBS, Roswell Park Memorial Institute (RPMI) medium, calcium chloride (CaCl_2_), and DMSO were purchased from Sigma-Aldrich (Switzerland) and used as received. *Staphylococcus aureus* (*S. aureus*) ATCC 25923, *Escherichia coli* (*E. coli*) ATCC 25922, and L929 mouse fibroblast cells were obtained from the Pasture Institute (Tehran, Iran).

### 2.2 Emulsion Preparation

The emulsion preparation used in this study was based on the method described in our previous study.^36^ The water-in-oil emulsion, consisting of an ALG aqueous solution in CHCl_3_, was prepared as follows: ALG was dissolved in distilled water at a concentration of 4% w/v by stirring for 4 h. Separately, Span as a suitable surfactant was dissolved in CHCl_3_ to obtain a 1 wt % solution to which the prepared ALG aqueous solution was added dropwise during 30 min under a high mixing speed of 20,000 rpm (Kinematica Polytron, PT2500 E, Switzerland). Finally, PCL granules were dissolved in CHCl_3_ as a continuous phase to obtain a concentration of 10% w/v. The volume ratio between the water phase and the oil phase was established at 0.1.

GEN as an antibacterial agent was added to the ALG aqueous solution at a concentration of 4 wt % (with respect to the total polymer weight). BTL as an antioxidant agent was dissolved in CHCl_3_ at concentrations of 5, 10, and 20 wt % (with respect to the total polymer weight). For confocal laser scanning microscopy (CLSM, LSM780, Carl Zeiss AG, Switzerland), fluorescein was added to the aqueous ALG solution.

### 2.3 Preparation of the Gentamicin-Loaded Alginate Hydrogel Film

To compare the drug release profile of GEN from the prepared core/shell nanofibers, a GEN-loaded alginate film was prepared as follows. An 8% (w/v) aqueous solution of ALG containing 4% (w/w) GEN was stirred for 8 h to create a homogeneous solution. A dropwise addition of a 0.1 M CaCl_2_ solution was then incorporated into the ALG solution with continuous stirring to achieve a final concentration of 36 mM. The resulting solution was then poured into a Petri dish and put at room temperature for 24 h to form hydrogel films.^37^

### 2.4 Electrospinning

To prepare core/shell ALG/PCL nanofibers, the prepared emulsion of ALG/PCL was loaded in a 3 mL plastic syringe (Braun, Germany) and a blunt needle (18G) was attached. A custom-built electrospinning setup was used,^38^ where a positive high voltage of 15 kV was applied on the needle and a negative voltage of 5 kV on the counter electrode, which was a flat aluminum plate. A feeding rate of 10 μL/min was maintained using a syringe pump (AL1000-220, WPI, USA), and the fibers were collected at a distance of 20 cm from the needle tip on aluminum foil. Also, drug-loaded PCL fibers were obtained using the aforementioned PCL solutions using similar parameters.

### 2.5 Characterization of the Morphology of Electrospun Nanofibers

The surface morphology of electrospun fibers was investigated using scanning electron microscopy (SEM; Hitachi S-4800, Hitachi High-Technologies, USA) at an accelerating voltage of 5 kV. Prior to SEM acquisition, the samples were sputter-coated with a 7 nm gold/palladium layer. The average diameter of fibers was measured based on 50 measurements using SEM micrographs using ImageJ software (USA).

The interior structure of electrospun fibers was further characterized by scanning transmission electron microscopy (STEM; Hitachi S-4800, Hitachi High-Technologies, USA) using an accelerating voltage of 30 kV. Samples for STEM were prepared by direct electrospinning onto copper grids placed on aluminum foil. To examine the cross section of the fibers, the nanofiber sample was first immersed in liquid nitrogen. After 5 min, it was transversely sectioned with a surgical blade. Subsequently, to extract the alginate from the structure, the sample was soaked in a water bath for 12 h. Post removal, a SEM image of the sample was captured. In addition, fluorescein-incorporated fibers that were directly electrospun on glass slides were used to evaluate the core/shell structure by CLSM using a 63× oil immersion objective at excitation and emission of 488 and 514 nm, respectively.

The infrared spectra of the samples were obtained using Fourier transform infrared spectroscopy (FTIR; BOMEM; Hartmann & Braun, Canada) by the KBr tablet method. The spectra were recorded in the range of 800–3800 cm^–1^ at a wavenumber resolution of 8 cm^–1^. The asymmetric stretching vibration peak height of C–H at 1937 cm^–1^ was used to normalize all the spectra.

The static water contact angle (WCA) of samples was measured using a Shape Analyzer DSA100 (Kruss, Germany) by the sessile drop method. The samples were put on the sample holder, and a droplet of deionized water was placed on the sample surface. The images were taken, and the contact angle was measured using the captured images.

### 2.6 Antibacterial Tests

The qualitative disk diffusion technique was employed to assess the antibacterial efficacy of the GEN-loaded core–shell nanofibers against both Gram-negative (*E. coli*) and Gram-positive (*S. aureus*) bacteria. To achieve this, a bacterial solution containing 1 × 10^5^ CFU/mL was introduced onto the agar dishes by using the spread method. Subsequently, the nanofiber samples were cut into 5 mm disks and positioned atop these plates. The plates were then subjected to an incubation period of 37 °C for 24 h, during which the emergence of an inhibitory region encircling each sample was gauged using three repetitions.

Additionally, the effectiveness of the core–shell nanofibrous structure on the antibacterial property over time was evaluated through an antibacterial touch test. For this purpose, both the GEN-loaded hydrogel film and nanofiber samples were cut into 5 mm disks and placed on agar plates. Subsequently, the plates were incubated for various time intervals: 5, 30, and 240 min. After the respective incubation periods, the disks were carefully removed, and the plates were further incubated at 37 °C for 24 h. Following this incubation period, the inhibition zones were measured for each sample.

### 2.7 *In Vitro* Drug Release Study

The release profile of GEN from the nanofibers was studied in PBS at a pH of 7.4. Standard solutions were prepared by dissolving known amounts of GEN in PBS. The calibration curve was plotted using the absorbance measurement of standard solutions at a wavelength of 256 nm using UV–vis spectrophotometry (UVmini-1240, Shimadzu, Japan) (Supporting Information, Figure S1). GEN-loaded nanofibers were cut, weighed, and incubated in 5 mL of PBS at 37 °C for different time intervals. A 1 mL solution was withdrawn from the release medium and replaced with fresh PBS. The obtained data were converted to the concentration using the standard curve.

The drug release kinetics of different GEN-loaded samples was investigated using the Peppas model (eq 1).^39^
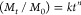
1where *t* is the time, *M_t_*/*M*_0_ is the drug fraction released at time *t*, *n* is the release exponent, which determines the release mechanism, and *k* is a constant depending on the geometry and structure of the system. To compare, the drug release profile from the GEN-loaded ALG hydrogel film was measured as well.

### 2.8 Encapsulation Efficiency

The encapsulation efficiency (EE%) of GEN-loaded samples was determined by dissolving weighed pieces of the GEN-loaded ALG hydrogel film in 5 mL of water and measuring the absorbance at λ_max_ = 256 nm. In the case of core–shell ALG/PCL samples, the PCL shell was dissolved in 5 mL of CHCl_3_. Then, 5 mL of water was added to the vial and shaken to dissolve remaining ALG and GEN. The resulting water phase was monitored at λ_max_, and the amount of GEN was calculated from the calibration curve. EE% was measured using eq 2, where *C* is the calculated weight of GEN and *C_t_* is the theoretical weight of GEN.
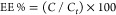
2

### 2.9 Antioxidant Activity Assay

The DPPH assay was carried out to investigate the antioxidant properties of electrospun samples with respect to BTL concentration and time according to the method of Banerjee et al.^40^ Briefly, 5 mg samples of BTL-loaded electrospun nanofibers were immersed in 3 mL of the 100 μM DPPH solution in methanol and incubated in the dark for 30 min. UV–vis spectrophotometry was used to measure the UV absorbance at 517 nm, and DPPH degradation was calculated according to eq 3, where *A*_C_ and *A*_S_ are the absorbance of positive control and sample at 517 nm, respectively.
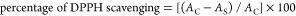
3

For the time-dependent assay, 5 mg of nanofiber membranes was exposed to 3 mL of 100 μM DPPH and the absorbance at 517 nm was recorded at different time intervals.

### 2.10 Cell Cytotoxicity

The viability of L929 mouse fibroblast cells on the core/shell ALG/PCL nanofibers was evaluated by utilizing the colorimetric MTT technique. The cells were cultivated in an RPMI medium supplemented with 10% fetal bovine serum (FBS) and 1% penicillin/streptomycin. This cell culture was maintained at a temperature of 37 °C with a 5% CO_2_ atmosphere within a CO_2_ incubator (APN-150, Padideh Nogen, Iran). Before the cells were introduced, the samples were sectioned into circular shapes with a diameter of 1 cm. These samples underwent sterilization under a UV lamp for a duration of 2 h. Following this, the samples were placed in 12-well plates along with cells at a density of 10^4^ cells per well. The coculture was maintained for a period of 24 h under conditions of 37 °C and 5% CO_2_.

Subsequent to the incubation, the wells underwent a PBS wash, following which a 100 μL MTT solution at a concentration of 5 mg/mL was introduced to each well. Following 4 h incubation at 37 °C, during which purple formazan crystals formed, the liquid medium was extracted. To dissolve the crystals, 200 μL of DMSO was added to each well. Ultimately, the concentration of color dissolved in DMSO was quantified using an ELISA plate reader (Sunrise, Bio Tek, USA), and the cell viability was subsequently computed.

### 2.11 Statistical Analysis

All measurements were made in triplicate, and the data were reported as the mean ± SD. One-way analysis of variance (ANOVA) was carried out to evaluate the result at the significance level of 0.05.

## 3 Results and Discussion

### 3.1 Characterization of the Electrospun Nanofibers

**Figure 1:**
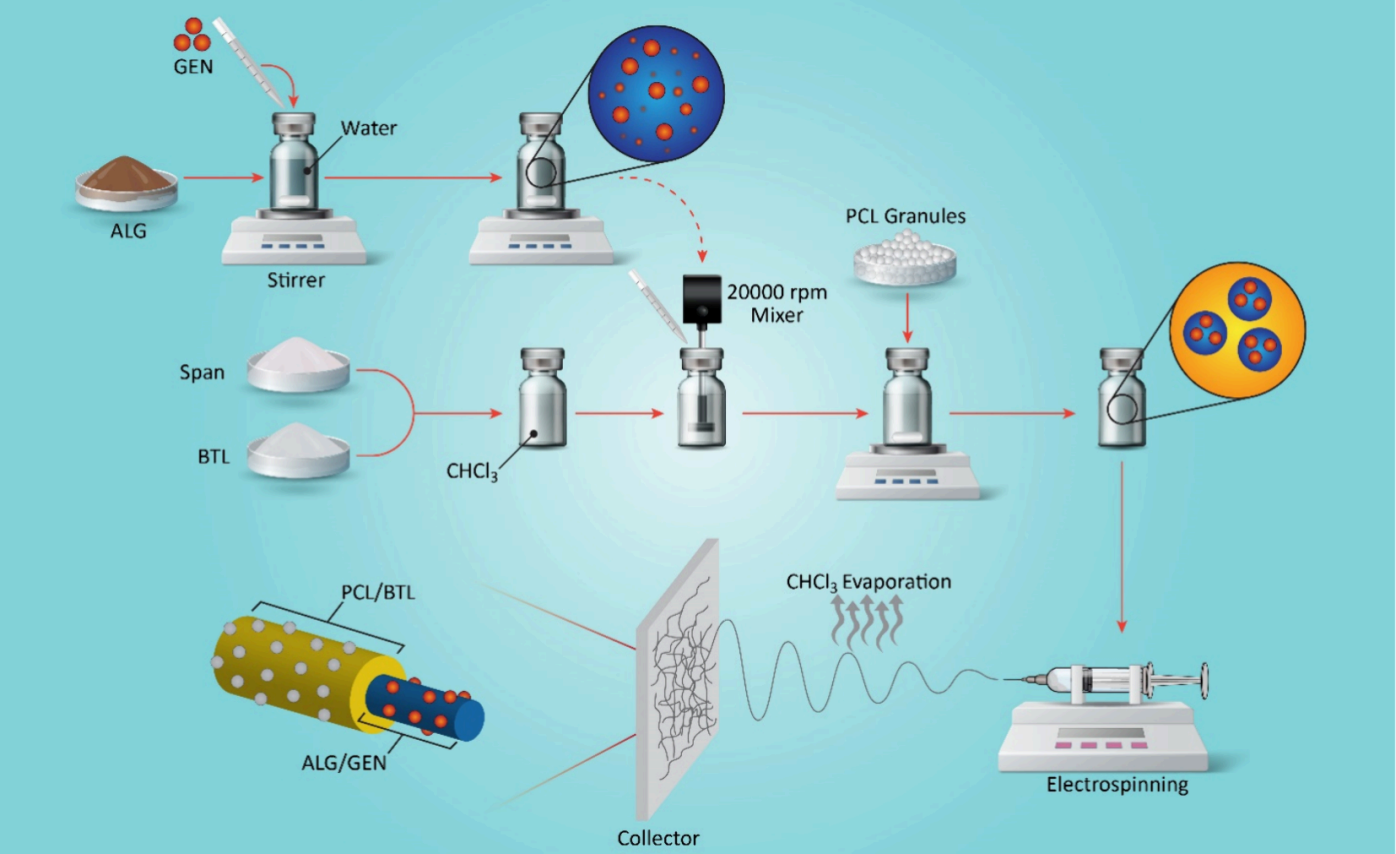
Schematic representation of the step-by-step fabrication method of core/shell ALG/PCL nanofibers loaded with therapeutic agents utilizing the emulsion electrospinning technique.

A stable water-in-oil (w/o) emulsion of ALG/PCL was prepared in this study. Two different agents were incorporated: GEN, an antibacterial and hydrophilic agent, was integrated into the aqueous phase of ALG, and BTL, an antioxidant and hydrophobic agent, was blended into PCL in CHCl_3_ as the continuous phase. Subsequently, core/shell ALG/PCL nanofibers were fabricated through emulsion electrospinning, with GEN encapsulated within the ALG core and BTL embedded in the PCL shell, as illustrated in Figure 1.

**Figure 2:**
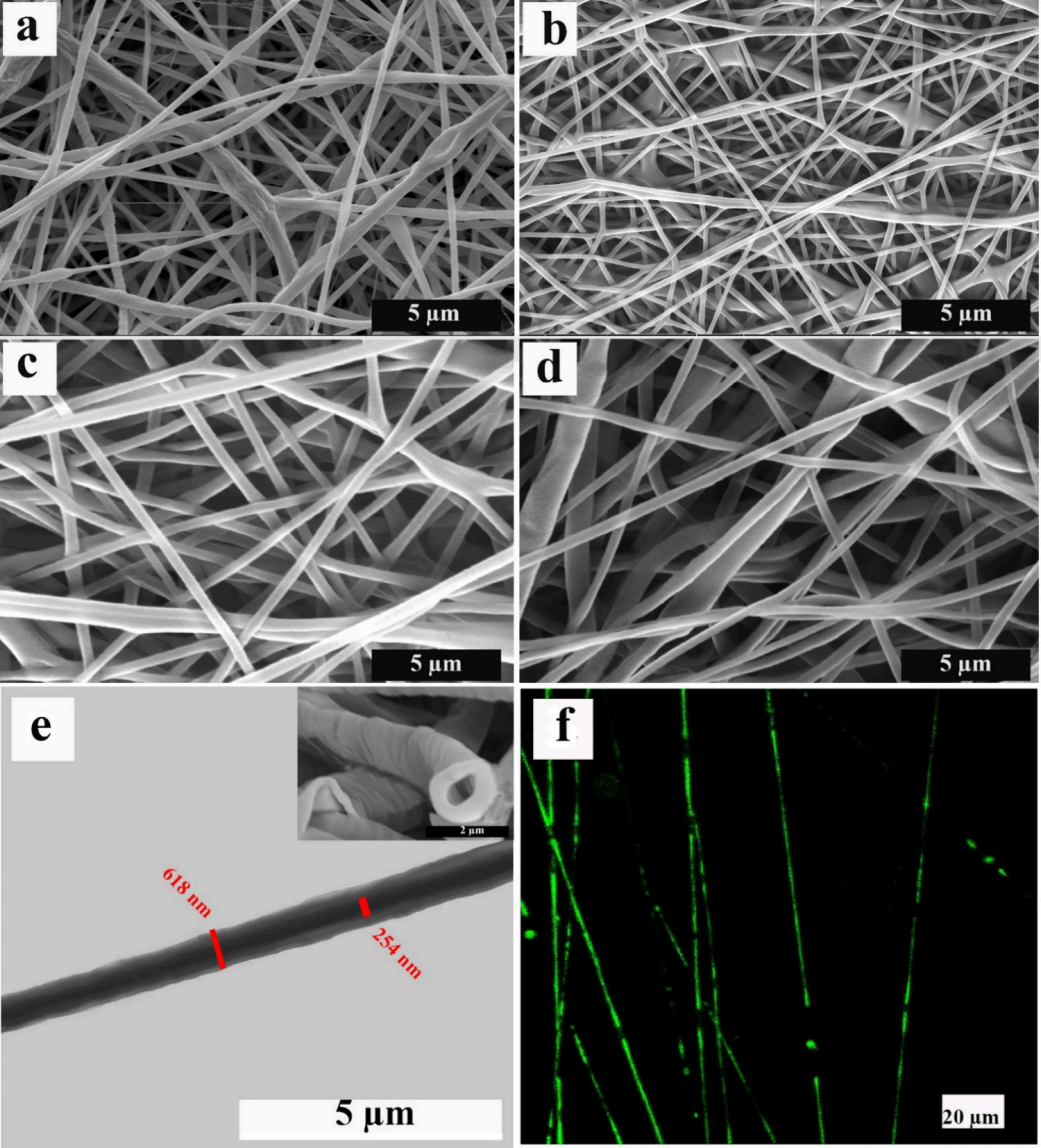
(a–d) SEM images of ALG/PCL nanofibers with different drug loadings: (a) B0G0, (b) B0G4, (c) B20G0, and (d) B20G4. (e) STEM and (f) CLSM images of the ALG/PCL nanofibers. The green channels in the CLSM image are fluorescein, which remark the ALG core of the nanofibers.

The details of prepared emulsions and the diameter of the relevant electrospun nanofibers are reported in Table 1, and the SEM, TEM, and CLSM images of the fabricated fibers are presented in Figure 2. Furthermore, the nanofibers’ diameter distribution histograms are shown in Figure S2 (see the Supporting Information). Bead-free nanofibers were obtained regardless of the emulsion composition. Furthermore, incorporation of GEN and BTL into the nanofibers had no considerable effect on the morphology or the diameter of the nanofibers.

**Table 1:** Specifications of Electrospun ALG/PCL Nanofibers

sample	BTL concentration (wt %)	GEN concentration (wt %)	mean diameter ± SD (nm)
B0G0	0	0	545 ± 118
B0G4	0	4	401 ± 109
B20G0	20	0	603 ± 117
B20G4	20	4	634 ± 223

To determine the inner structure of the resulting nanofibers, STEM was performed (Figure 2e). The contrast in the STEM images demonstrates the core–shell structure in the resulting electrospun fibers, in which darker and brighter regions are assigned to the core and shell, respectively. Additionally, the figure’s top section displays the cross-sectional image of the fibers, illustrating the removal of the alginate core subsequent to immersion in a water bath. The diameters of the nanofiber and the ALG core were measured, and they are included in the image.

Aiming to further investigate the core continuity in the electrospun fibers, fluorescein was loaded in the core solution, and CLSM images were taken as displayed in Figure 2f. These images remark the continuity of the core in the ALG/PCL nanofibers. Nevertheless, the weakness of the detected green color of fluorescein at some points is obvious, which is assigned to the interruption of coalescing of ALG-dispersed droplets during the electrospinning process.

To investigate the hydrophilicity and chemical composition of core/shell ALG/PCL nanofibers, WCA and FTIR measurements were performed. It was observed that WCA values for PCL and B0G0 nanofibers are within a similar range (see the Supporting Information, Figure S3), which reveals almost alike hydrophobicity for both PCL and B0G0. This indicates that PCL is mostly located in the outer layer of ALG/PCL nanofibers. Also, no significant difference was observed between WCA data for the rest of the samples, i.e., B0G4, B20G0, and B20G4 with PCL nanofibers (*p* > 0.05). Throughout the electrospinning process, PCL chains in the continuous phase experience high strain. Consequently, aqueous ALG droplets as the dispersed phase begin coalescing with each other along the spinning direction. Meanwhile, CHCl_3_ as an organic solvent in the continuous phase evaporates faster than water in the dispersed phase, so the viscosity in the continuous phase increases significantly. Thus, the outer layer of the fiber solidifies faster than the dispersed phase. This difference in viscosity between the organic matrix and the dispersed aqueous phase results in an inward movement of the ALG-dispersed droplets and, subsequently, their coalescence. Finally, the hydrophobic PCL forms the shell and the dispersed ALG droplets are transferred into the core, resulting in core/shell electrospun fibers.^41,42^

**Figure 3:**
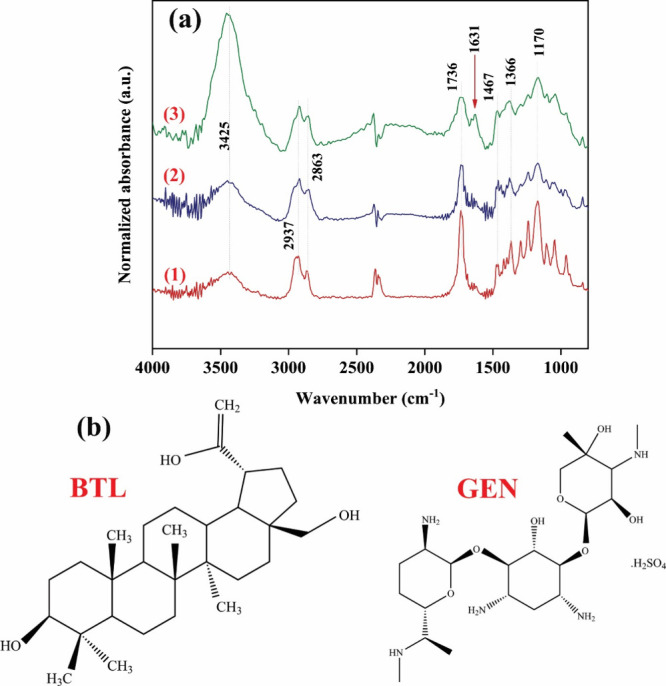
(a) FTIR spectra of (1) PCL, (2) B0G0, and (3) B20G4 electrospun fibers. (b) Chemical structures of BTL and GEN.

FTIR spectra of PCL, B0G0, and B20G4 electrospun nanofibers in the wavenumber region of 800–4000 cm^–1^ are presented in Figure 3a. With respect to the PCL spectrum, characteristic peaks of the C–H asymmetric and symmetric stretching vibration, C=O stretching vibration, C–H bending vibration, and C–O stretching vibration are observed at 2937, 2863, 1736, 1467, 1366, and 1170 cm^–1^, respectively.^43−45^ Considering the B0G0 spectrum, the appearance is similar to the PCL spectrum. However, the height ratio of the hydrogen-bonded O–H stretching signal at 3425 cm^–1^ to the C=O stretching vibration signal at 1736 cm^–1^ increased significantly. This could be explained by the existence of ALG polymer chains within the B0G0 core, which feature hydroxyl groups along their backbone and potentially absorbed water molecules.

Looking at the B20G4 spectrum, the magnitude of the prominent peak observed at 3425 nm exhibits a noteworthy augmentation. This enhancement can be attributed to the abundance of oscillations arising from organic moieties, specifically emanating from the stretching vibrations of amino groups within gentamicin (Figure 3b). Additionally, the intensified presence of hydrogen-bonded hydroxyl groups in BTL further contributes to this heightened effect.^46^ In addition, the B20G4 spectrum displayed a new peak at 1631 cm^–1^, which can be assigned to the C=C stretching of BTL (Figure 3b).^47^

### 3.2 Drug Release Study

**Figure 4:**
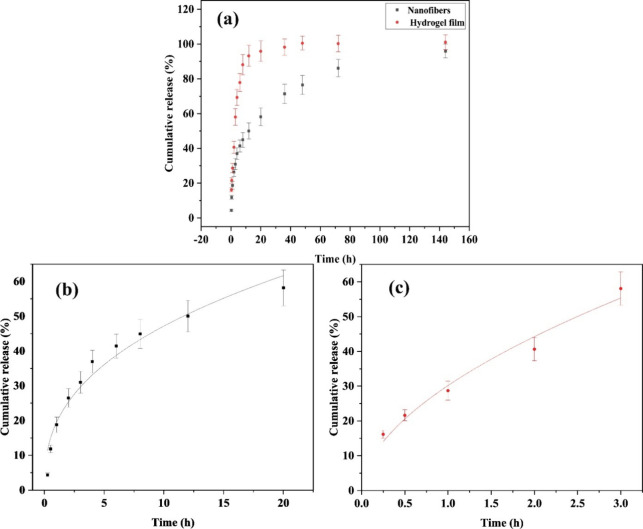
GEN release profile from core/shell ALG/PCL nanofibers and ALG hydrogel film at a drug loading percent of 4% (a). Experimental data points (symbols) and predicted (solid lines) data from the Peppas model fitted on the 60% portion of the drug release curve of nanofibers (b) and hydrogel film (c).

The drug release behavior of GEN-loaded core/shell ALG/PCL nanofibers and ALG hydrogel film was investigated. The calculated EE% of samples is reported in Table 2. The drug release behavior of the samples is illustrated in Figure 4. Two distinct release stages of the drug could be observed for two samples including a burst release (stage I), which continues with a steady-state (stage II) drug release. The drug release from the core/shell nanofibers was found to be slower than that from the hydrogel film. After 12 h, the cumulatively released drug reaches 93% in the hydrogel film sample, while in the case of nanofibers, this value decreased to 50%. Overall, nanofibers showed lower burst release, implying the efficacy of this structure compared to the hydrogel film.

**Table 2:** EE% and Peppas Parameters of Different GEN-Loaded Samples

			Peppas parameters	
sample	theoretical GEN loading (wt %)	EE%	*R*^2^	*n*
nanofibers	4%	53.3 ± 5.8	0.96	0.38
hydrogel film	4%	67.1 ± 6.5	0.97	0.55

To investigate the drug release mechanism from the samples, the Peppas model (eq 1) was fitted to the release data (60% portion of the release), and the calculated parameters are reported in Table 2. Considering the *n* values, GEN release from the nanofibers followed the Fickian diffusion mechanism. It means that the GEN release mechanism is mainly attributed to diffusion or permeation. Considering the slow degradation of PCL in an aqueous environment, it was expected that the diffusion mechanism dominates the drug release over this period of time. In contrast, the *n* value for the ALG hydrogel film is 0.55, indicating anomalous diffusion, in which the diffusion process cannot be explained using Fick’s law.

GEN is characterized as a hydrophilic molecule due to the presence of hydroxyl and amine groups. Given the hydrophilic nature of ALG, arising from its hydroxyl and carboxyl groups, there exists a favorable compatibility between GEN molecules and the ALG matrix. This compatibility extends to the context of the ALG hydrogel films. Nevertheless, in the case of ALG/PCL nanofiber samples, GEN molecules must navigate through the PCL shell to access the surface. Consequently, it is expected that GEN will exhibit a more controlled release profile from ALG/PCL nanofibers in comparison with ALG hydrogel films.

### 3.3 Antibacterial Properties

**Figure 5:**
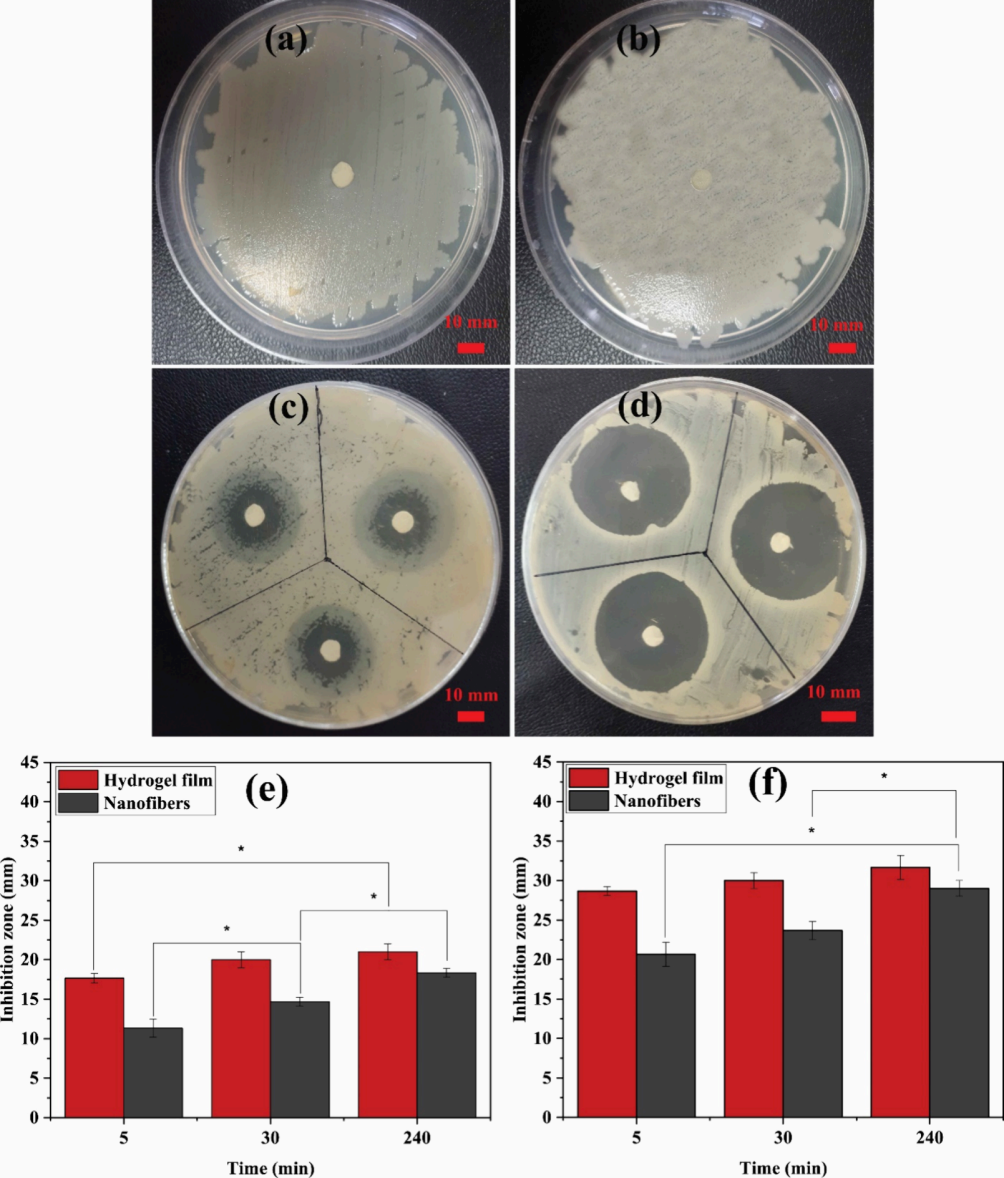
Inhibition zone of ALG/PCL (a, b) and GEN-loaded ALG/PCL (c, d) nanofibers against *E. coli* (a, c) and *S. aureus* (b, d) after 24 h of incubation. Comparison of antibacterial properties of the GEN-loaded hydrogel film and ALG/PCL nanofiber samples against (e) *E. coli* and (f) *S. aureus*. Columns identified with a star are significantly different (*P* < 0.05).

The antibacterial properties of core/shell ALG/PCL nanofibers were carried out using an antibacterial disk diffusion test in triplicates. The inhibition zones of control samples and 4% GEN-incorporated ALG/PCL nanofibers against Gram-negative *E. coli* and Gram-positive *S. aureus* are shown in Figure 5. Upon 24 h of incubation, noticeable sensitivity was observed in both strains toward GEN-loaded nanofibers. *E. coli* and *S. aureus* exhibited inhibition zones of approximately 22 ± 1 and 31 ± 1 mm, respectively. The bigger inhibition zone against *S. aureus* marks the higher sensitivity of this strain against GEN than *E. coli*.

A comparison of antibacterial properties of the GEN-loaded hydrogel film and ALG/PCL nanofiber samples was carried out using an antibacterial touch test at different time intervals of 5, 30, and 240 min against Gram-negative *E. coli* and Gram-positive *S. aureus* to evaluate the impact of the core/shell nanofiber structure (see the Supporting Information, Figure S4). Figure 5e,f presents the corresponding bar chart. It can be observed that the nanofiber samples exhibited smaller inhibition zones compared to those of the hydrogel film samples at each time interval. This can be attributed to the slower release of GEN from the nanofibers. Additionally, the difference in inhibition zones between each time interval was more pronounced for the nanofiber samples against both strains compared to the hydrogel film samples. These findings highlight the potential of the core/shell ALG/PCL nanofibers in controlling the release profile of GEN and decreasing the initial burst release, as confirmed by the UV drug release study, as well.

### 3.4 Antioxidant Activity

**Figure 6:**
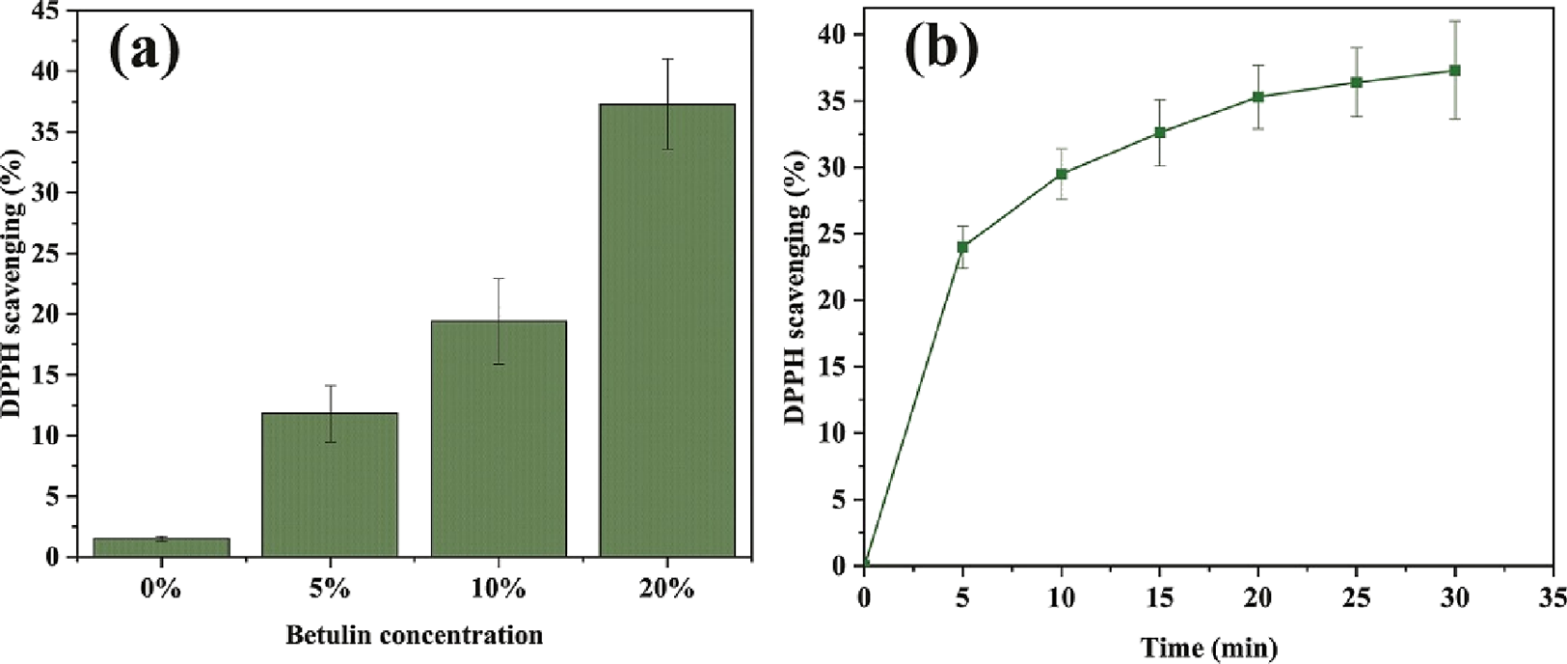
(a) DPPH scavenging efficiency of core/shell ALG/PCL nanofibers at different concentrations of BTL incorporation after 30 min. (b) Time-dependent antioxidant activity of 20% BTL-incorporated core/shell ALG/PCL nanofibers.

Antioxidant properties of core/shell ALG/PCL nanofibers were studied by a DPPH assay. DPPH is a stable free radical that exhibits maximum absorption at 517 nm. When accepting an electron or hydrogen atom, DPPH free radicals can be neutralized, resulting in a color change of the solution from purple to yellow.^48^ According to Figure 6a, the DPPH scavenging efficiency of nanofibrous samples depends on the concentration of incorporated BTL. Increasing the BTL concentration results in higher DPPH scavenging efficiency, which means higher antioxidant activity, whereas incorporation of 20% BTL reveals 37.3% DPPH scavenging.

The time-dependent antioxidant activity of 20% BTL-incorporated nanofibers is depicted in Figure 6b. Although DPPH scavenging increases over time, the slope of the curve decreases. This marks the accomplishment of the reaction, which indicates that there are no more electrons or hydrogen atoms to quench DPPH free radicals.

A research study has shown that high levels of reactive oxygen species (ROS) contribute to impaired wound healing.^49^ Decreasing ROS is believed to accelerate wound healing by stimulating cell migration and angiogenesis.^50^ A persistent inflammatory response in chronic wounds results in ROS accumulation more than the antioxidant capacity of the cells. This phenomenon hinders the phase change of the wound from inflammatory to proliferation.^51^ Thus, keeping the balance of redox in cells is expected to prevent abnormal cell growth and the disturbance of immune response.^50^ Therefore, introduction of the antioxidant function to wound dressings is expected to accelerate wound healing, especially in chronic wounds. In an innovative study, He et al. developed a multifunctional nanofibrous wound dressing by electrospinning PCL and quaternized chitosan-*graft*-polyaniline (QCSP), which demonstrated enhanced wound healing in a mouse model. The PCL/QCSP membrane, in particular, showed promising results in antibacterial and antioxidant activity, cell proliferation, and accelerated healing, with increased collagen deposition and angiogenesis observed.^52^

### 3.5 Cytotoxicity Assay

**Figure 7:**
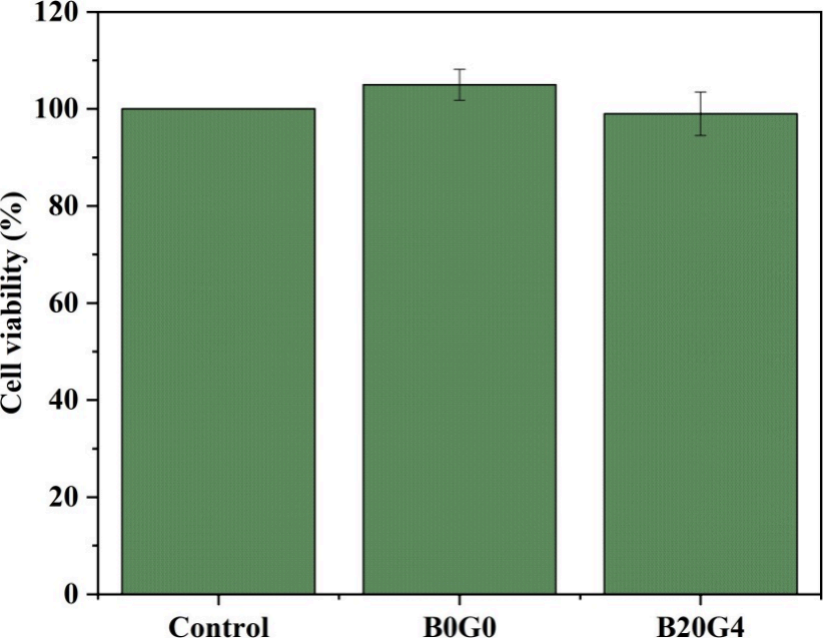
Cell viability of L929 mouse fibroblast cells exposed to nanofiber samples.

The adhesion and growth of L929 mouse fibroblast cells on both B0G0 and B20G4 samples were evaluated through the MTT assay, as depicted in Figure 7. This technique quantifies the conversion of the MTT tetrazolium compound into a purple formazan compound by viable cells. Consequently, the degree of MTT reduction, which signifies regular mitochondrial activity, can be correlated with the level of cellular metabolism. As anticipated, no harmful effects on cell viability were detected in connection with the nanofiber samples. Moreover, there were no notable distinctions observed between cell proliferation on these samples and the control group.

Numerous studies have been conducted on the incorporation of therapeutic agents within core/shell nanofibers.^23,53,54^ In their pivotal study, Maleki et al. explored the fabrication of core/shell nanofibers using a coaxial electrospinning system specifically designed for drug delivery applications. The research focused on utilizing tetracycline hydrochloride (TCH) as the core material and poly(lactide-*co*-glycolide) for the shell. A comparative analysis was conducted between the drug release profiles of monolithic fibers produced by blend electrospinning and the core/shell structures. The findings highlighted the superior efficacy of the core/shell morphology in controlling drug release.^23^ In another notable study, Yan et al. successfully fabricated poly(vinyl alcohol)/chitosan (PVA/CS) core/shell nanofibers through a coaxial electrospinning technique. These core/shell fibers demonstrated potential as carriers for the delivery of doxorubicin (DOX), a chemotherapeutic agent. *In vitro* cytotoxicity tests further revealed the biocompatibility of the core/shell fibers, with the encapsulated DOX exhibiting lower cytotoxicity compared to free DOX.^54^ It is acknowledged that the incorporation of therapeutic agents into core–shell nanofibers has been the subject of extensive research. However, our work distinguishes itself through the novel incorporation of BTL and GEN within the nanofiber matrices. This study is the first to report the simultaneous encapsulation of both hydrophilic GEN and hydrophobic BTL agents in a single nanofiber system, leveraging the unique properties of each to enhance the therapeutic efficacy. Furthermore, the technique employed in our study, emulsion electrospinning, represents an innovative approach to the field. This method has enabled us to achieve stable incorporation of these agents, which has not been previously demonstrated. The dual delivery system, crafted via emulsion electrospinning, offers significant benefits for applications that demand a broad-spectrum antibacterial impact, complemented by controlled release kinetics and bolstered by appropriate antioxidant activity. However, one potential limitation of our nanofibers could be the scalability of the emulsion electrospinning process for mass production. Future work will focus on optimizing the fabrication process to enhance the practicality of our nanofibers for commercial use. Additionally, while our nanofibers show promising biocompatibility in cell culture studies, further *in vivo* studies are necessary to fully understand their interaction with complex biological systems and their long-term biodegradability in a clinical setting.

## 4 Conclusions

This work aimed to obtain antibacterial–antioxidant core–shell ALG/PCL nanofiber membranes. In this regard, GEN as a hydrophilic antibiotic and BTL as a hydrophobic natural compound were incorporated into the ALG core and PCL shell, respectively. The core–shell fiber membranes were generated via emulsion electrospinning using a single needle setup. STEM and CLSM images indicated the core/shell structure of the electrospun fibers, further confirmed by WCA measurement, indicating that PCL is mostly located on the surface of ALG/PCL nanofibers. An *in vitro* drug release study showed the Fickian diffusion mechanism for the GEN-loaded nanofibers. Also, decreasing the burst release and slower release of GEN from core/shell nanofibers compared to the ALG hydrogel film was observed during this study. The antibacterial test confirmed the suitable antibacterial property of 4% GEN-loaded nanofibers against both Gram-negative and Gram-positive *E. coli* and *S. aureus*, respectively. The DPPH assay specified that the DPPH scavenging efficiency of the nanofibers depends on the amount of BTL that was incorporated. Moreover, the cytotoxicity assay indicated no cytotoxic effect on the nanofibers. In comparison to existing materials for wound dressings, our core–shell nanofibers demonstrate a novel approach by effectively encapsulating and controlling the release of both hydrophilic and hydrophobic drugs. While traditional materials may offer either rapid drug release or sustained release, our nanofibers are engineered to minimize the initial burst release of hydrophilic drugs such as gentamicin, which is a common drawback in current systems. This controlled release profile is achieved without compromising the antibacterial efficacy or the antioxidant properties, as evidenced by our *in vitro* tests. Overall, the here fabricated antibacterial–antioxidant core/shell nanofibrous membranes show great potential as wound dressings with dual functions to prevent infection and accelerate the wound healing process.

## Supporting Information Available

Calibration curve of GEN in PBS at a wavelength of 256 nm (Figure S1); fiber diameter distribution histograms of ALG/PCL nanofibers with different drug loadings (Figure S2); water contact angle values for different electrospun nanofibers (Figure S4); inhibition zone of the GEN-loaded hydrogel film and nanofiber samples against *S. aureus* and *E. coli* at two time points of 5 and 240 min (Figure S4) (PDF)

## Notes

^∥^ This author passed away in June 2020.
