# Supplementary material for: Dual Functional Antibacterial–Antioxidant Core/Shell Alginate/Poly(ε-caprolactone) Nanofiber Membrane: A Potential Wound Dressing
Source: ACS Omega. 2024 May 30;9(23):25124–34. doi: 10.1021/acsomega.4c02510 (PMC11170714; doi:10.1021/acsomega.4c02510)
Supplement: Supplementary file 1 — ao4c02510_si_001.pdf [file ao4c02510_si_001.pdf]

Dual functional antibacterial-antioxidant core/shell alginate/ poly(ε-caprolactone) nanofibers membrane: A potential wound dressing

Mohammad-Reza Norouzi[1,2], Laleh Ghasemi-Mobarakeh[2,*], Fabian Itel[1], Jean Schoeller[1,3], Hossein Fashandi[2], Giuseppino Fortunato[1,†], René M. Rossi[1,3,*]

[1]Empa, Swiss Federal Laboratories for Materials Science and Technology, Laboratory for Biomimetic Membranes and Textiles, Lerchenfeldstrasse 5, CH-9014 St. Gallen, Switzerland

[2]Department of Textile Engineering, Isfahan University of Technology, Isfahan 84156-83111, Iran

[3]ETH Zürich, Department of Health Science and Technology, 8092 Zürich, Switzerland

* Corresponding authors' email addresses: laleh.ghasemi@iut.ac.ir, rene.rossi@empa.ch

†this author passed away in June 2020

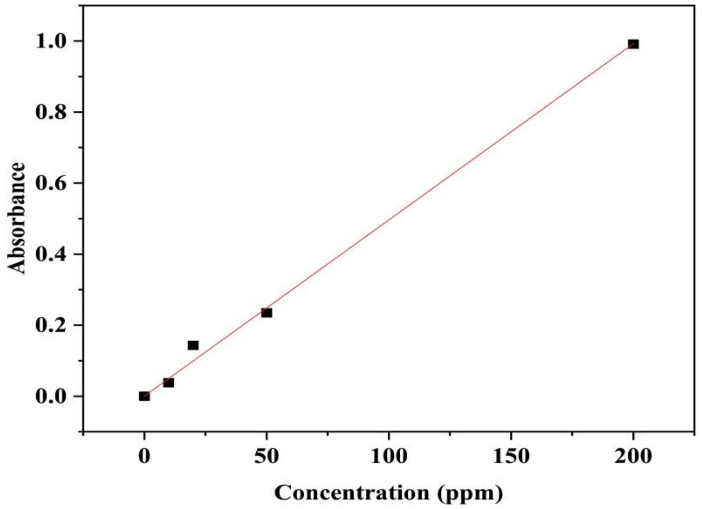

Figure S1. Calibration curve of GEN in PBS at wavelength of 256 nm.

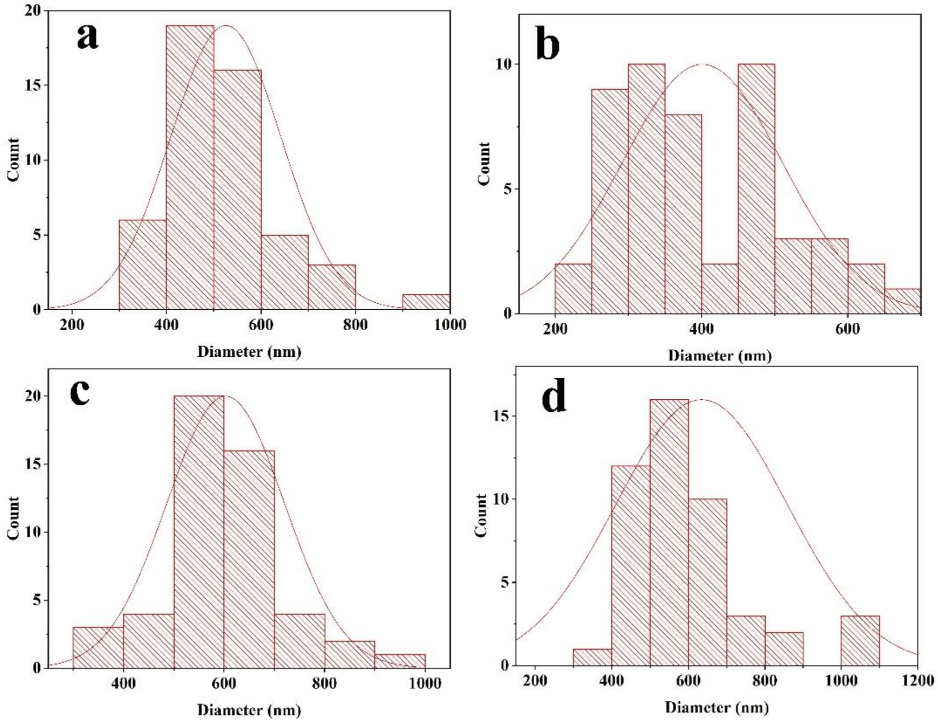

Figure S2. Fiber diameter distribution histograms of ALG/PCL nanofibers with different drug loadings: (a) B0G0, (b) B0G4, (c) B20G0 and (d) B20G4.

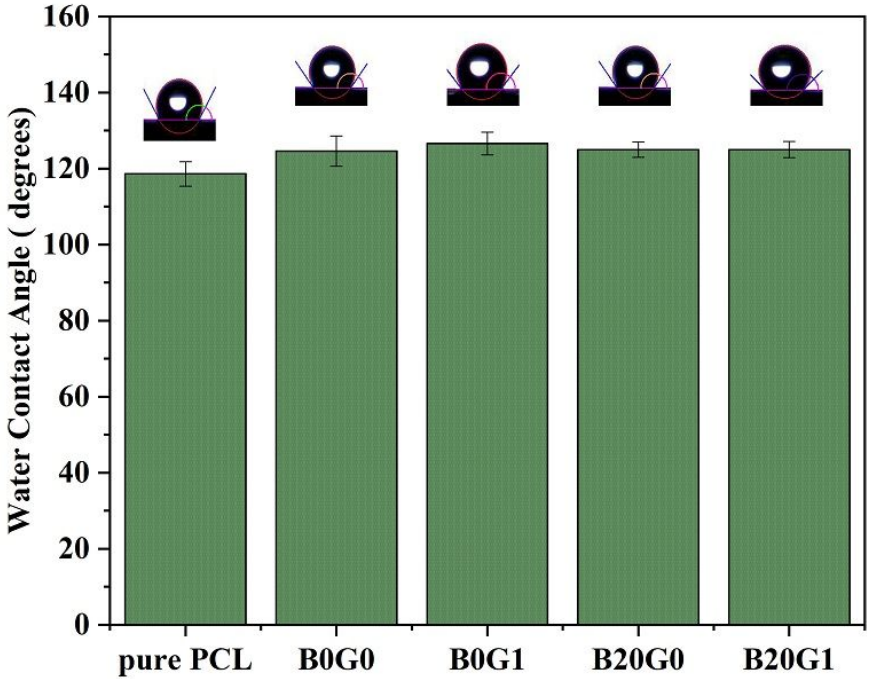

Figure S3. Water contact angle values for different electrospun nanofibers.

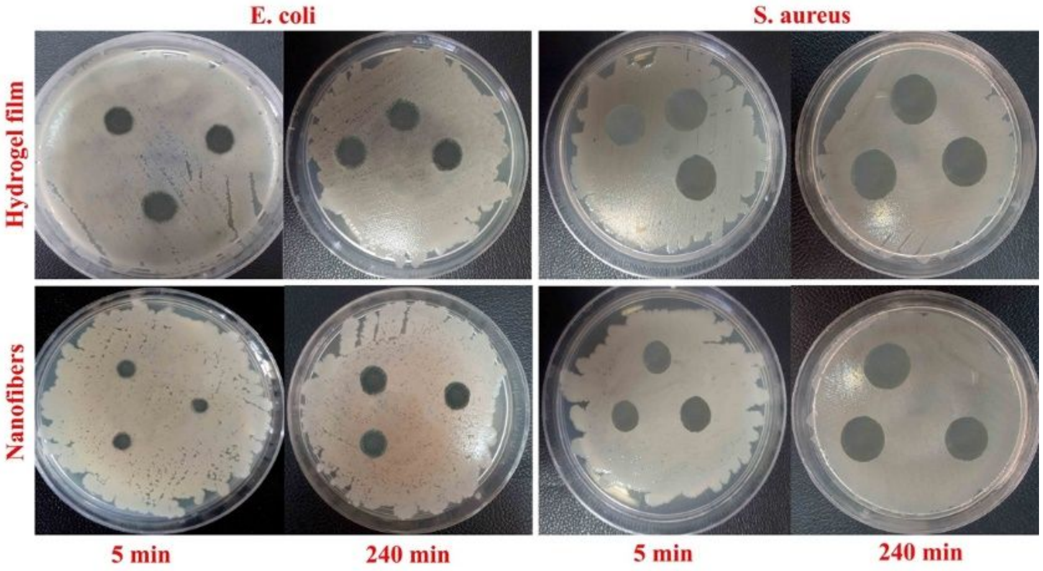

Figure S4. The inhibition zone of GEN-loaded hydrogel film and nanofibers samples against *S. aureus* and *E. coli* at two time-point of 5 and 240 min.
